# Application of a Platform for Gluten-Free Diet Evaluation and Dietary Advice: From Theory to Practice

**DOI:** 10.3390/s22030732

**Published:** 2022-01-19

**Authors:** Gesala Perez-Junkera, Maialen Vázquez-Polo, Francisco Javier Eizagirre, Laura Benjumea, Carlos Tutau, Blanca Esteban, Jonatan Miranda, Idoia Larretxi, Virginia Navarro, Itziar Churruca, Arrate Lasa

**Affiliations:** 1 Gluten3S Research Group, Department of Nutrition and Food Science, Faculty of Pharmacy, University of the Basque Country UPV/EHU, 01006 Vitoria-Gasteiz, Spain; gesala.p@gmail.com (G.P.-J.); maialen.vazquez@gmail.com (M.V.-P.); jonatan.miranda@ehu.eus (J.M.); idoia.larrechi@ehu.eus (I.L.); virginia.navarros@ehu.eus (V.N.); arrate.lasa@ehu.eus (A.L.); 2 Bioaraba, Grupo Nutrición y Seguridad Alimentaria, 01009 Vitoria-Gasteiz, Spain; 3 Department of Pediatric Gastroenterology, Hospital Universitario Donostia, 20006 San Sebastián, Spain; FRANCISCOJAVIER.EIZAGUIRREAROCENA@osakidetza.eus (F.J.E.); LAURA.BENJUMEAMORENO@osakidetza.eus (L.B.); 4 Department of Pediatric Gastroenterology, Hospital Universitario de Cruces, 48903 Barakaldo, Spain; carlos.tutaugomez@osakidetza.eus; 5 Asociación de Celiacos y Sensibles al Gluten de Madrid, 28028 Madrid, Spain; consultasdieta@celiacosmadrid.org; 6 Centro Integral de Atención a Mayores San Prudencio, 01006 Vitoria-Gasteiz, Spain

**Keywords:** digital platform, celiac disease, gluten-free diet, digitalized dietary assessment, nutritional education

## Abstract

The present work aimed to analyze, through the *GlutenFreeDiet* digital platform, the evolution over one year of the nutritional status, dietary profile and symptoms present among celiac people on a gluten-free diet (GFD) while receiving individualized dietary advice. Twenty-seven adults and thirty-one celiac children/adolescents participated in the study. This was then followed up by three visits, at diagnosis, and after 3 and 12 months (vt0, vt3 and vt12). Participants filled out dietary and gastrointestinal symptoms questionnaires. All patients received written personalized dietary advice from dietitians who interpreted data from the platform. Results obtained indicated that participants consumed proteins and lipids in excess and carbohydrates in defect. Low intakes of cereals, fruit and vegetables and high meat intakes were observed. However, gluten-free product (GFP) consumption and that of ultra-processed foods was reduced after 1 year in adults. Symptoms decreased after vt3 but recurred in vt12. Changes in ultra-processed foods and GFP intake, but lack of changes in the rest of the parameters suggested that the platform support was not effective enough. Even though the platform represents a useful tool for monitoring celiac patients and giving dietary advice, modules that require more continuous attention and nutritional education of patients should be provided for interventions to be more effective.

## 1. Introduction

Digital devices have changed the way we relate to others, including medical services. What is more, the current pandemic has given value to all those forms of communication that allow us to bring knowledge, news or people closer without leaving the safety of our homes. There are many advantages to using this type of tool [1]. On the one hand, its permanent presence in people’s lives means that symptoms, changes in habits or any data of interest can be recorded immediately. Moreover, the clinician could have access to these data at the very moment of their introduction. On the other hand, due to the extended use of electronic devices among the population, access to eHealth platforms is widespread, and people who would otherwise have had less access through other means of communication, could be reached. However, these applications still have a long way to go, since issues such as the analysis of the results obtained in patients using these tools have been approached unevenly and with results that should be interpreted with caution [2,3]. In fact, studies on these types of eHealth applications are becoming increasingly frequent [2].

In the current digital market, there are numerous platforms with very interesting applications for different pathologies or in pandemic diseases such as obesity or diabetes. Most of them are digital platforms, also available as an app, with visual, clear and accessible information. In the case of celiac disease (CD), there are several applications, mainly designed for celiac people and with different purposes: apps to find restaurants and grocery stores that cater to GFDs; apps to quickly check the ingredients on food labels; apps for discovering gluten-free recipes, or apps designed to inform people about what gluten is, where can it be found and how it can be avoided. However, there are no available platforms that give personalized information about the dietary profile or the health and nutritional status of celiac people, which in fact is a matter of concern, since GFD patients need to guarantee not only the total elimination of gluten from the diet but also the fulfilment of all dietary recommendations for each individual.

When nutritional assessments of celiac people have been carried out, imbalanced proportions of macronutrients and several vitamin and minerals deficiencies have been observed in their diets [4,5,6,7,8,9]. In particular, these studies have confirmed that a GFD is usually characterized by a poor intake of carbohydrates, fibre, iron, calcium, folate, niacin and zinc, as well as an excessive consumption of saturated fats. Therefore, following a GFD could lead to an increased risk of suffering from several pathologies related to dietary imbalances, such as cardiovascular diseases [10], anemia, osteoporosis or constipation [11,12,13]. Indeed, clinical trials performed among celiac participants have observed altered biochemical parameters, which are related to the aforementioned associated pathologies [7,14,15,16,17].

Therefore, and in view of this scenario, a nutritional intervention based on personalized nutritional advice and a close follow-up of celiac patients carried out by dietitian nutritionists (DN) should be highly recommended in order to establish suitable dietary guidelines. In fact, authors working in this field have proposed a regular monitoring of dietary history, apart from measurements of serum antibodies, body composition and examination of symptoms related to nutritional deficiencies, as a strategy for improving the nutritional status of celiac sufferers and for making GFD more balanced [18]. E-health devices could represent a good tool for achieving this goal.

Nevertheless, professionals working with this population do not have suitable and available electronic devices for an appropriate evaluation of a GFD and they can only make approximations. Platforms for GFD design and evaluation that are available on the market do not include nutritional information about a large number of specific GFPs, which have been demonstrated to be different from those of their gluten-containing homologues [19,20]. Thus, the diet of people with CD cannot be precisely designed and their nutrient intake is usually miscalculated. Moreover, most commercial software focuses on dietary plan designs for healthy people and are not freely available. Only a small number of these programs are also useful for therapeutic dietary plans, including diseases such as diabetes, hypercholesterolemia, or hypertension—but not CD. 

The Gluten3S research group designed the free open digital *GlutenFreeDiet* for design and evaluation of GFD (http://www.ehu.eus/dieta-singluten/ (last accessed 20 December 2021) [21]. This platform contains different sections, which process clinical data of celiac people: anthropometric data, biochemical parameters, dietary habits and symptom presence. For dietary evaluation, apart from analysing the nutritional composition of all conventional foods, this device details the composition of more than 700 gluten-free rendered foodstuffs, including breads, muffins, cereals, biscuits, etc. Therefore, the software allows dietitians to measure GFD’s energy content and nutrient distribution exactly, in addition to the impact of specific GFPs on total energy intake. This fact is of the utmost importance because many authors have suggested that the imbalance of GFD could be due to the contribution of GFPs, which seems to be higher in celiac children than in adults [22,23,24]. 

It must be also taken into account that nutritional imbalances are not the only difficulty faced by people with CD. Gluten elimination from diet should lead to a total remission of symptoms. However, data in the literature indicate that approximately 20–30% of celiac people continue suffering from symptoms even though they follow a strict GFD [25,26,27,28,29]. One of the reasons attributed to these complications is the involuntary ingestion of small amounts of gluten, called dietary transgressions [27]. There are several factors which can cause this, including a lack of knowledge about both gluten-containing and non-containing products, the high price of GFP, sharing meals with non-CD diners [30], socialization problems of the celiac people or anxiety and depression [31,32,33,34,35]. Thus, ensuring adherence or a strict compliance to GFD has proved to be crucial not only for a faster improvement of intestinal lesion but also for the reduction of symptoms related to the CD and the normalization of body composition [36]. Another possible cause for symptom maintenance has been attributed to other molecules present in the diet, apart from gluten, and which are known to be harmful in other digestive diseases such as Irritable Bowel Syndrome. This is the case of fermentable oligo-, di- and monosaccharides and polyols (FODMAPs), present in several foods from derived from plants, dairy products and sweeteners. It has been observed that when dietary FODMAP intake is reduced, symptoms such as abdominal pain, bloating, diarrhea or constipation diminish [37,38,39,40]. Thus, measuring and controlling their consumption could also make sense in CD. The platform *GlutenFreeDiet* gives the option to measure both dietary adherence and FODMAP consumption. 

Taking into account all of the above, it is obvious that this group needs a suitable platform for nutritional assessment, education and follow-up. Thus, the aim of this study was to evaluate the implementation of the *GlutenFreeDiet* platform among patients with CD for one year, in order to monitor and evaluate the evolution of their nutritional status, dietary habits and presence of symptoms, and to assess its usefulness as a future eHealth utility.

## 2. Materials and Methods

### 2.1. Participants and Procedure

**Figure 1:**
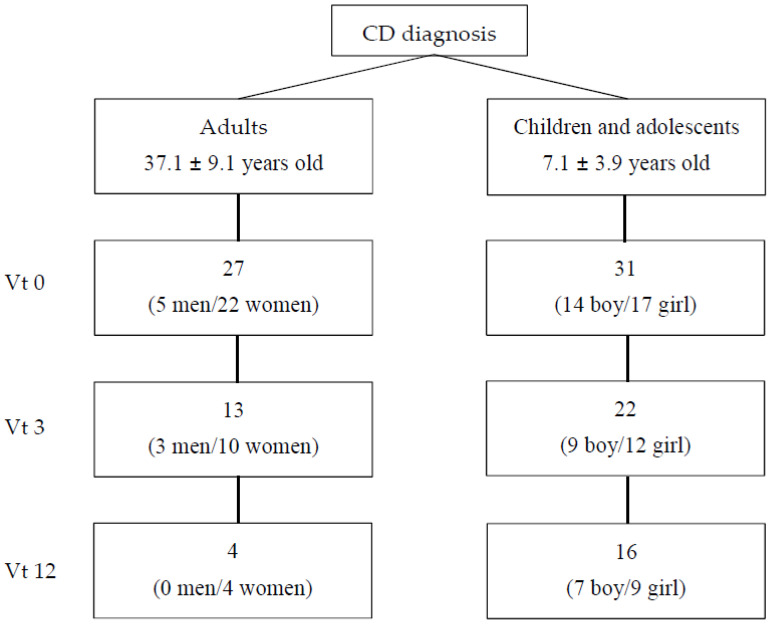
Patients included in the study. Vt0: visit at time 0, at diagnosis; vt3: visit after 3 months on a gluten-free diet; vt12: visit after 12 months on a gluten-free diet.

A study group of celiac people was recruited between 2016 and 2018 in two hospitals in the Basque Country (Hospital Universitario de Cruces and Hospital Universitario de Donostia) and in the Celiac Association of Madrid (Asociación de Celiacos y Sensibles al Gluten de Madrid). All pediatric patients (n = 31) were referred to the clinics to confirm a CD diagnosis over this time span (2016–2018) and were consecutively enrolled and followed for the first 12 months after diagnosis confirmation. In the case of adults, who were taken from Celiac Association of Madrid members, 10 participants were newly diagnosed and 17 had been on a GFD for less than a year. All participants attended medical/dietitian offices on three occasions: at diagnosis (vt0); after three months on a GFD (vt3); and after 12 months on a GFD (vt12). Twenty-seven adults (5 men, 22 women; mean age ± SD: 37.1 ± 9.1) and thirty-one celiac children and adolescents (mean age ± SD: 7.1 ± 3.9) took part in the study at vt0; thirteen adults (three men and 10 women) and twenty-two children and adolescents continued the study at vt3 and four adults (all women) and sixteen children and adolescents at vt12 (Figure 1). All available data were used.

Exclusion criteria included a history of chronic diseases such as cardiovascular disease, diabetes, hyperthyroidism/hypothyroidism, hypercholesterolemia, hypertriglyceridemia or high blood pressure levels, and other digestive pathologies that need specific dietary advice, as well as a lack of motivation to participate in the study. Informed written consent was obtained from all participants after receiving information about the survey. This study was approved by the Ethical Committee of The Basque Country (Comité Ético de Investigación Clínica de Euskadi (CEIC), Code PI 2016069).

### 2.2. Anthropometric Measurements

Trained personnel collected anthropometric measurements. Body weight (±10 g) was measured using a digital integrating scale (SECA 760; SECA, Hamburg, Germany). Height was determined to the nearest 5 mm using a stadiometer (SECA 220; SECA, Hamburg, Germany). Body Mass Index (BMI) was calculated from weight and height (kg/m^2^). The BMI values of adult patients were categorized according to the World Health Organization (WHO) criteria as follows: Below 18.5 kg/m^2^ considered as underweight, 18.5–24.9 kg/m^2^ as normal weight, 25–29.9 kg/m^2^ as overweight and >30 kg/m^2^ as obese (WHO). In the case of children and adolescents, the criteria established by Sobradillo et al. [41] were used to categorize BMI values as follows: <percentile 3 was considered as underweight; between percentile 3 and 85 normal weight, between percentile 85 and 95 overweight and >percentile 95, obese. All these data were introduced in the platform *GlutenFreeDiet*, in the “Anthropometry” section.

### 2.3. Body Composition and Energy Expenditure

Fat mass, muscle mass, water, protein and mineral body content were estimated by a direct segmental multiple-frequency bioelectrical impedance analysis method (Inbody 120; Microcaya, S.A., Bilbao, Spain). Two skin electrodes were placed on the feet and hands. Following the standard procedure, whole-body resistance and reactance were measured. The guidelines of WHO [42,43] and Moreno et al. [44,45] were used as reference for body fat mass in adults and children respectively. Muscle mass values were compared to the limits described by Heymsfield et al. [46] and Ito et al. [47]. Protein, mineral and water content for each participant were classified according to the limits established by InBody 120.

Resting metabolic rate (RMR) and waist and hip circumferences (WHC) were also calculated by the bioelectrical impedance analysis method. In order to calculate energy expenditure, standard activity level value was applied to the RMR. The limits defined by WHO were used to classify waist-height ratio data [48]. All these data were introduced in the platform *GlutenFreeDiet*, in the “Anthropometry” section.

### 2.4. Biochemical Data

Fasting glucose, total cholesterol, HDL-cholesterol (HDL-c), LDL-cholesterol (LDL-c), triglycerides, ferritin and transferrin levels were measured at each visit. Values were compared to the Basque Health System references. All these data were introduced in the platform *GlutenFreeDiet*, in the “Biochemical Data” section.

### 2.5. Analysis of Symptoms Presence

For the systematic evaluation of current gastrointestinal symptoms, participants filled out a self-administered, structured Gastrointestinal Symptom Rating Scale (GSRS) questionnaire [49]. This is a validated questionnaire used widely in research on CD and other gastrointestinal disorders [50,51,52,53,54,55]. The questionnaire measures five sub-dimensions of gastrointestinal symptoms: indigestion, diarrhea, abdominal pain, reflux and constipation. It comprises 15 separate items altogether. Values for each of the five sub-dimension scores were calculated as a mean of the respective items and the total GSRS score as a mean of all 15 items. Scoring is based on a Likert scale from 1 to 7 points, where 1 point means minimal gastrointestinal symptoms and 7 points the most severe symptoms. Total scores of all participants were divided into three groups: 0 answers marked; 1–5 answers marked out of 15; and 6 or more symptoms marked out of 15. All these data were introduced in the platform *GlutenFreeDiet*, in the section of “Questionnaires”, specifically under the Symptoms Questionnaire.

### 2.6. Dietary Assessment and Counselling

Dietary intake was assessed using 3-day 24-h food recalls (24 HR) in each visit to the medical/dietitian office (vt0, vt3 and vt12), two on weekdays and one at the weekend. Participants also filled out a food frequency questionnaire (FFQ) at the first and last visit (vt0 and vt12). Nutritionist dietitians supported participants in answering the questionnaires and recorded data. In order to avoid bias in the measurement of the diet, food portions and amounts were determined by using photographs of rations and sizes described in Rusolillo and Marques´ Photo Album [56]. Energy and nutrient intakes were calculated through the *GlutenFreeDiet* platform (“24 h recall” section) [21]. 

When the platform was designed, Dietary Reference Intakes (DRI) for the Spanish population issued by the Spanish Societies of Nutrition, Feeding and Dietetics (FESNAD) in 2010 were taken as references for the interpretation of the 24 HR [57]. In the case of FFQ, the Spanish Society of Community Nutrition (SENC) recommendations were used for the correct interpretation of the results [58].

In addition, energy contribution of GFP was calculated for each participant and the food or drink items were classified using the NOVA system. This system is the most commonly used for the identification and definition of ultra-processed foods in scientific literature [59]. NOVA categorizes foods according to their nature, purpose and degree of industrial processing [60] and it distinguishes between the following groups: (i) unprocessed or minimally processed foods, (ii) processed culinary ingredients, (iii) processed foods and (iv) ultra-processed products. 

All data processed by the platform *GlutenFreeDiet* were summarized in a personalized written form and sent to each participant after each visit to the medical/dietitians office (after vt0, vt3 and vt12). These reports detailed their nutritional status diagnosis and the quality of their diet, such as their consumption of macronutrients, food groups, their micronutrient deficiencies and possible associated risks, etc. Moreover, personalized dietary guidelines were provided to each patient in order to correct particular imbalances. Patients had the option of requesting personalized consultations concerning their results through the platform, but most of them did not use this service.

### 2.7. Determination of FODMAP Daily Intake 

Quantification of daily intake of total fructan, galacto-oligosaccharides (stachyose and raffinose), polyols (sorbitol and mannitol), excess of fructose (calculated subtracting glucose content to fructose content) and lactose, was performed. With the exception of fructans, FODMAP concentrations (g per 100 g of food) of general foods were obtained from the open-access Food Composition Database named Food Standards Australia New Zealand [61]. As this Australian database does not provide information about total fructan (only inulin), research articles were used as a reference for total fructan content of vegetables and fruit [62], as well as for pulses [63]. In the cases of cereal-based foodstuffs, fructan content data were obtained from our own determinations following a methodology previously described [64]. Data on FODMAP content were uploaded to the platform GlutenFreeDiet and the consumption of these compounds was calculated through this digital device [21].

### 2.8. Statistical Analysis

Statistical analyses of results were performed by using the IBM SPSS statistical program, version 23 (IBM Inc., Armonk, NY, USA). Normality in the distribution was assessed by the Kolmogorov-Smirnov test, and homogeneity by Levene’s test. Statistical analyses were performed in order to calculate differences between measurements using Wilcoxon test (vt0 vs. vt3 and vt0 vs. vt12) and one way ANOVA (adults vs. children). Correlation between variables was calculated with Pearson´s correlation coefficient test. Variables with skewed distribution were logarithmically transformed to obtain a more symmetrical distribution. *p* values < 0.05 were accepted as statistically significant.

## 3. Results

### 3.1. Anthropometric and Biochemical Parameters over 1 Year of GFD in Adults and Children with CD

The evolution of anthropometric and biochemical data among adult participants and children over one year on GFD is presented in Supplementary Table S1. All parameters from all participants were under normal values at the beginning and no changes were observed after 1 year on a GFD.

### 3.2. Energy, Macronutrient, Fibre and Cholesterol Intake of Adults, Children and Adolescents with CD 

Table 1 shows the energy intake, macronutrient distribution, and fibre and cholesterol consumption of adults and children. Energy intake was in accordance with the energy expenditure of participants. All of them (adults and children) showed a bad distribution of macronutrients in their diets, which was characterized by an excess of protein and lipids and a low consumption of carbohydrates. Saturated fatty acid intake was high among adults and children, whereas consumption of sugars was adequate in all participants. Data obtained after 3 and 12 months on GFD did not indicate a modification of this dietary profile, with the exception of protein consumption among children, which increased after 3 months on GFD but turned to vt0 values after 1 year.

Fibre intake was insufficient at vt0 in both groups but it increased after 3 months on a GFD. In fact, participants complied with fibre recommendations in vt3 and vt12. Mean cholesterol intake was below maximum intake recommendation only in Vt0 for all groups. 

### 3.3. Food Frequency Consumption 

The daily consumption of vegetables, fruit, oils and especially that of cereals was low at vt0 in all participants (Table 2) and, more specifically, cereal consumption among children showed a tendency toward decreased values (*p* = 0.098) after 12 months on a GFD. Meat consumption was extremely high in all subjects, exceeding twice the recommended intake. 

A year on a GFD with dietary advice reduced the frequency of meat consumption in adults and children, reaching statistical significance in the latter group. However, the decrease observed was not enough to fulfil recommendations. The intake of the rest of food groups remained unchanged after the intervention. 

Regarding the differences between groups at each moment of the intervention, whereas children ate lower quantities of vegetables than adults, the opposite results were observed for legumes, where recommendations were only fulfilled by children. Consumption of pastries was higher in children than in adults during the whole intervention, but only reached statistical significance at vt0.

### 3.4. Food Consumption According to NOVA System

NOVA classification indicated that during the follow-up, all participants increased the intake of foods included in group 1 (unprocessed or minimally processed foods) and decreased that of foods from group 4 (ultra-processed products) (Table 3). 

When data between adults and children were compared, statistical differences were observed in the consumption of foods from group 1 at vt0 and vt3, from group 2 at vt3, from group 3 at vt0 and from group 4 at vt3 and vt12, indicating that adults consumed, in general, more natural foods and less ultra-processed foods. In order to obtain a deeper insight into the most consumed ultra-processed foods at each visit, a list showing the percentage of consumption of each food group from G4 was performed (Supplementary Table S2). The major contribution was observed for GFP in all visits.

### 3.5. GFP Contribution to the Total Energy Intake

Table 4 shows GFP energy contribution to the total calorie intake of participants, which was higher in adults at the beginning of the intervention. This contribution decreased among adults during the intervention and did not change among pediatric participants. The specific contribution of each GFP (bread, pasta, cake/scone/bun/biscuit, cereals, cookies, pizza, cereal bars and fajita/crepe/pancakes) was also analyzed and results indicated that bread was the main contributor. 

### 3.6. FODMAP Consumption of Celiac Adults and Children

Total FODMAP consumption increased after 3 months on a GFD among adult participants (Table 5). This increase in adults could be due to increases in glucose, fructose and inulin. Even though these three FODMAPs also showed increased values in vt3 and vt12 among children, they were not enough to observe an increase in the total amount of FODMAPs consumed. Differences between adults and children were observed at the beginning but not at the end of the intervention.

### 3.7. Presence of Gastrointestinal Symptom 

**Figure 2:**
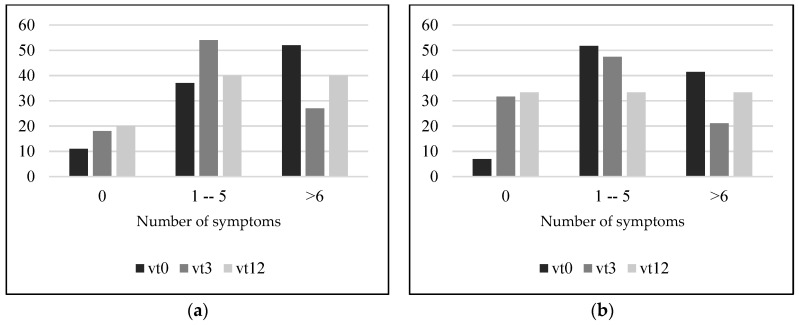
Percentage of adult (**a**) and children and adolescent (**b**) participants presenting 0, 1–5 or more than 6 symptoms. Vt0: visit at time 0, at diagnosis; vt3: visit after 3 months on a gluten-free diet; vt12: visit after 12 months on a gluten-free diet.

Symptom presence evolution indicated that in both adults and children, symptom presence decreased after 3 and 12 months on a GFD (Figure 2a,b) because the number of participants that presented no symptoms increased after 3 and 12 months on a GFD. Similarly, 3 months on a GFD led to a reduction in the number of patients with more than six symptoms at vt0. However, the number of patients with more than six symptoms increased again between vt3 and vt12 in both populations (adults and children). These changes in symptom presence only reached statistical significance in the case of children and between the visits vt0 and vt3 (*p* = 0.059).

## 4. Discussion

It is known that CD is a common form of malabsorption [65,66,67]. For the time being, a lifelong rigorous GFD is the only available treatment effective in remitting the symptoms of CD. Nevertheless, symptoms can sometimes remain even though patients go on a GFD. Moreover, this dietary pattern can result in an imbalanced proportion of macro- and micronutrient ingestion in both minors and adults with CD [4,22,68]. Several reasons have been proposed to justify these results, such as the scarce nutritional education of this group or the lack of a strict compliance to the diet [6,36]. Thus, nutritional counselling and a regular follow up by DN to celiac and gluten-sensitive people have been proposed as key strategies for achieving successful results in both removing symptoms and dietary balance [6,18] and, as a consequence, in the nutritional status and quality of life of this population. Digital platforms could represent a suitable tool for this regular follow-up of celiac patients since they are available in electronic devices used daily and thus the way to regularly access a large number of patients. However, there is no specific dietary software for CD because most of them do not include specific GFP in their databases and the ones that do, only contain a small range of these foodstuffs. 

The *GlutenFreeDiet* platform represents a potential eHealth tool for assessing the dietary profile and other health parameters of celiac people, for example, body composition, biochemical data or symptomatology-. This open and free platform, available at http://www.ehu.eus/dieta-singluten/ (accessed on 20 December 2021) can be also downloaded to mobile devices from the platform itself and can be used by both health professionals working in the field of CD, and celiac patients interested on their nutritional status and dietary pattern. The present study shows the implementation of the abovementioned platform, a pilot nutritional intervention through this digital device within a segment of the celiac community. 

Other nutritional interventions published in the literature indicate that the dietary pattern of celiac people on GFD is not balanced. Even though energy consumption can adjust to energy waste, macronutrient distribution is not balanced. Protein and lipids (especially saturated ones) are usually consumed in excess among people following a GFD, and by contrast, carbohydrate consumption is low [5,6,69,70]. It has to be considered that GFP have partly been blamed for this imbalance, for several reasons: the high price of GFP (source of carbohydrates) that cause celiac sufferers to avoid them [71]; cereals used in GFP manufacturing that are poorer in proteins and usually not whole grains [19,72], and the use of fats and gums in their production in order to improve palatability [19], etc. In fact, our previous studies have identified these nutritional composition differences between GFP and their gluten-containing counterparts [19]. Nevertheless, in the present study, apart from GFP consumption, there were other dietary factors that altered energy distribution, such as the dietary habits of the subjects. 

Regarding fibre intake, it was noted as being low among adults at diagnosis, whereas it became appropriate at vt3 and vt12. This could be due to several factors. On the one hand, to an increase in fresh foods from plants, such as fruit or vegetables, which even though these were not changed in the FFQ, a tendency toward increased values of the unprocessed foods (group 1 according to NOVA classification) could indicate more abundance of fibre sources in the diet. Moreover, although GFP consumption decreased at vt3 among adults, it did not among children, and it has been observed that fibre content does not differ between GFP and their homologues as much as macronutrient content does. In fact, it has been recently noted that gluten-free breads in particular can contain even more fibre than those containing gluten, suggesting that the food industry is making efforts to raise the content of this nutrient in GFP [73,74,75].

As mentioned above, poor consumption of cereal, fruit, vegetables and oils was observed in all groups of participants, data that is in accordance with the literature [4,9,22,68,76]. By contrast, meat was consumed in excess, which could be linked to the greater contribution of saturated fats in the diet at diagnosis. In fact, a positive correlation between both parameters was observed among celiac children at diagnosis (*p* = 0.02). Likewise, this nutrient consumption decreased slightly among children at vt3 and vt12, as did meat consumption. In the light of the above, giving dietary advice after vt0 and the follow-up in vt3 and vt12 did not change participants’ dietary pattern totally, but it was effective for a reduction in meat consumption. 

The intake of ultra-processed foods has been widely linked to a greater cardiometabolic risk and mortality [77,78,79,80] and should thus not only be avoided among the general population but also among people with CD [81,82]. Therefore, the reduction of these kind of foods should also be another goal of all nutritional interventions performed in the celiac people. In the present study, food consumption was classified according to the NOVA system, a reference method for the identification and definition of ultra-processed foods. All participants, both adults and children, reduced the consumption of these foods by the end of the intervention, and by contrast, increased that of unprocessed foods. Therefore, it could be suggested that even though positive effects of the intervention were not globally observed for the whole dietary profile, it was effective in reducing the consumption of ultra-processed foods. 

The contribution of GFP to the total energy intake has been also analysed in several studies and mainly among children—due to the fact that this population usually eats more cereal derivatives such as biscuits or breakfast cereals. In our previous studies, in addition to those of other authors, around 27 and 36% of the total energy intake of celiac children going on a GFD for more than 1 year came from GFP [22,23]. In the present study, this percentage was much lower in both children and adults, and besides, it was reduced among adults during the intervention. These differences between the present study and those already published underline the importance of monitoring celiac people from the beginning of the diagnosis and giving nutritional education relative to GFP. 

Another aim of the introduction of a GFD is the partial or total elimination of symptoms [1,83,84]. Nevertheless, the period required for symptom remission varies between individuals [85,86] and there is a small proportion of patients that continue suffering from symptoms even though they follow a GFD [86,87]. In the present study, an amelioration of gastrointestinal symptoms in the first three months was observed among children, which was probably due to the motivation of starting the intervention and thus to a complete initial adherence to the diet. However, after this period a return of symptoms was observed in vt12, which indicated the probable relaxation of these participants in strictly following the GFD. In fact, the literature also indicates that dietary adherence to GFD can decrease with the duration of treatments [88,89] and the greater the level of adherence, the more effective the GFD is in resolving symptoms [90,91]. Symptoms reported by participants as having decreased at the beginning but recurring by the end of the intervention, indicate that the concern about both the diet and the presence of symptoms was not as important at the end as at the beginning. Unfortunately, dietary adherence among the participants was not collected in the present study. In this respect, Silvester et al. have shown that almost all patients with CD only manage to maintain a gluten-reduced diet but not totally gluten-free [92]. Thus, these data suggest that a continuous follow-up of patients is necessary for ensuring the total adherence to the diet and for obtaining its beneficial results in symptom absence, not only in the short term, but also in the long term.

Other causes for the appearance of gastro-intestinal symptoms in CD are being investigated, such as the consumption of FODMAPs. The benefits of removing these compounds from a diet has been widely demonstrated in several diseases such as the Irritable Bowel Syndrome [37,93]. As far as we know, this is the first time that exact FODMAP consumption is being measured among recently diagnosed celiac patients. Data obtained indicated no changes of FODMAP ingestion during the intervention, or, as observed among adults, an increased consumption in vt12. Therefore, the observed reduction in symptoms cannot be linked to a reduction in FODMAPs among participants of the present study. 

Considering all the abovementioned factors, it is clear that correct personalized dietary counselling, nutritional education and a continuous follow-up of celiac and gluten sensitive patients performed by DN is crucial for obtaining positive results. Digital platforms could represent a useful tool not only for processing data, but also for providing the mentioned nutritional assessment and advice. In the present study, positive results were obtained since meat or ultra-processed food consumption decreased throughout the intervention. However, a lack of changes in the rest of the dietary pattern suggests that the intervention was not effective enough. In an attempt to find a simple form of counselling, which is not too stressful for the patient and is feasible for healthcare personnel, patients were given personalized dietary advice through individualized reports, which probably resulted in a lack of complete adherence, disinformation or failing to grasp the guidelines. It also appears that reports did not capture the interest of the participants. Although they had the option of requesting personalized consultations through the platform, most of them did not do so. Furthermore, the number of participants who dropped out during the course of the study was substantial. For more successful results, a continuous and more frequent monitoring in addition to patient nutrition education should be carried out while applying a more effective methodology, namely: integrating in the platform *GlutenFreeDiet* new options such as educational tools for patient empowerment (forums, webinars, workshops, educational games and others) as well as versatile communication channels for a closer contact with the patient (WhatsApp and email attention, messages or notifications, regular phone or video calls, etc.). Bearing this in mind, this study represents a pilot experience which will lead to a second improved intervention study—to start in the near future—in order to achieve better outcomes and to provide an effective eHealth instrument.

The main limitation of the present study was the high number of participants that failed to continue with the study during the follow-up and the consequent lack of data at vt3 and vt12. Thus, a second improved study, as well as any further studies aimed to examine GFD evolution in celiac people, must overcome this limitation. Moreover, it would be interesting to analyze vitamin and mineral consumption or to quantify, in addition to fructans, the rest of FODMAPs of GFP. However, it is worth highlighting the long period in which participants were monitored in the present study, as well as the amount of data collected about the evolution of their nutritional status, symptom presence and dietary habits including data such as ultra-processed foods, GFP and FODMAP intake, which were all processed by the platform *GlutenFreeDiet*. It is also worth noting that this study provides relevant information about decisive aspects that future interventions should address.

## 5. Conclusions

The platform *GlutenFreeDiet* represents a useful device for giving personalized and continuous nutritional assessment and advice by DN to people with CD, and thus, to achieve improvements in their dietary habits. This could lead to a positive effect on the evolution of their pathology, their nutritional status and, as a consequence, on their quality of life. However, nutritional advice given solely via individualized reports during the first year on a GFD was only effective in correcting some aspects of the dietary pattern, but not all of them. Therefore, a combination of closer counselling and continuous attention via a platform is required, where general nutrition education together with the provision of individualized information may serve as an encouragement for patients.

## Figures and Tables

**Table 1:** Energy and macronutrient daily intake of celiac adults and children (mean ± SD).

		Adults					Children and Adolescents					Adults vs. Children		
	Recommended Intake *	vt0	vt3	vt12	*p* Value		vt0	vt3	vt12	*p* Value		*p* Value		
**N**		27	13	2	vt0 vs. vt3	vt0 vs. vt12	22	10	4	vt0 vs. vt3	vt0 vs. vt12	Vt0	Vt3	Vt12
**Energy intake (kcal)**	±20% of EE	1968.4 ± 607.4	2079.7 ± 474.0	1944.0 ± 97.7	NS	NS	1867.5 ± 517.4	2194.2 ± 393.5	1944.0 ± 97.7	NS	NS	NS	NS	NS
**Protein (%)**	12.5	16.3 ± 3.6	19.6 ± 3.6	19.8 ± 2.2	NS	NS	16.3 ± 3.7	20.3 ± 3.5	19.8 ± 2.0	<0.05	NS	NS	NS	NS
**Lipids (%)**	32.5	41.1 ± 7.8	36.6 ± 6.7	40.6 ± 4.6	0.08	NS	41.3 ± 7.4	36.0 ± 7.3	40.6 ± 4.6	NS	NS	NS	NS	NS
**Saturated fatty acids (%)**	<10	12.8 ± 6.8	12.1 ± 2.8	14.5 ± 2.6	NS	NS	12.0 ± 4.3	12.8 ± 2.9	14.5 ± 2.6	NS	NS	NS	NS	NS
**Carbohydrates (%)**	55	40.5 ± 8.3	42.5 ± 7.0	36.3 ± 7.1	NS	NS	40.2 ± 8.3	42.7 ± 8.0	36.3 ± 7.1	NS	NS	NS	NS	NS
**Simple sugars (%)**	<10	4.3 ± 2.2	3.7 ± 1.2	4.1 ± 0.1	NS	NS	4.6 ± 2.3	3.8 ± 1.1	4.1 ± 0.1	NS	NS	NS	NS	NS
**Fibre (g)**	14 g/1000 kcal (25–35 g)	21.6 ± 11.2	26.3 ± 11.6	25.3 ± 7.1	0.1	NS	19.2 ± 10.0	25.9 ± 9.3	25.3 ± 7.1	0.06	NS	NS	<0.05	NS
**Cholesterol (mg)**	<300	298.8 ± 132.3	302.7 ± 77.0	378.3 ± 125.2	NS	NS	296.3 ± 138.9	326.5 ± 65.5	378.3 ± 125.2	NS	NS	NS	NS	NS

Abbreviations: Vt0 = visit at time 0, at diagnosis; vt3 = visit after 3 months on a gluten-free diet; vt12 = visit after 12 months on a gluten-free diet; EE = Energy Expenditure. *p* < 0.05: Statistically significant; NS = Not Significant. * Recommended contribution to a balanced diet proposed by the Federation of Spanish Societies of Nutrition and Dietetics (FESNAD) and Spanish Society for Community Nutrition (SENC) [57,58].

**Table 2:** Food group consumption frequency of celiac adults and children that took part in the study (mean ± SD).

			Adults			Children and Adolescents			Adults vs. Children	
		Recommended Daily Portion Intake *	vt0	vt12	*p* Value	vt0	vt12	*p* Value	*p* Value	
**N**			22	4	vt0 vs. vt12	31	16	vt0 vs. vt12	vt0	vt12
**Daily consumption**	Dairy	2–4	2.6 ± 1.4	3.2 ± 0.3	NS	2.7 ± 1.3	2.5 ± 0.6	NS	NS	NS
	Cereals	4–6	2.6 ± 1.4	2.7 ± 0.8	NS	3.8 ± 1.9	2.7 ± 1.5	0.098	<0.05	NS
	Vegetables	2	1.3 ± 1.4	2.0 ± 1.3	NS	0.8 ± 0.5	0.9 ± 0.6	NS	<0.05	<0.05
	Fruits	3	2.3 ± 1.6	2.2 ± 1.5	NS	1.7 ± 1.3	2.0 ± 1.1	NS	NS	NS
	Oils	3–6	2.5 ± 1.2	2.7 ± 2.3	NS	2.1 ± 1.4	2.4 ± 1.3	NS	NS	NS
**Weekly consumption**	Meat	3–4	6.2 ± 2.5	4.5 ± 1.3	0.1	7.2 ± 3.5	6.5 ± 1.4	<0.01	NS	NS
	Fish	3–4	3.5 ± 2.3	4.3 ± 1.4	NS	3.1 ± 1.6	3.4 ± 1.6	NS	NS	NS
	Eggs	3–4	2.9 ± 2.5	3.0 ± 1.4	NS	2.7 ± 1.2	2.8 ± 0.6	NS	NS	NS
	Legumes	2–4	1.9 ± 1.3	1.7 ± 1.2	NS	2.6 ± 1.2	2.5 ± 0.9	NS	0.06	0.1
	Nuts	3–7	2.6 ± 2.2	2.2 ± 1.4	NS	1.3 ± 2.1	1.1 ± 1.2	NS	NS	NS
	Pastries	Ocassional (<1 portion/week)	0.8 ± 0.9	0.3 ± 0.3	NS	1.3 ± 1.7	1.8 ± 2.2	NS	<0.05	NS

*p* < 0.05: Statistically significant; NS = Not Significant. * Recommended intake according to the Spanish Society of Community Nutrition (SENC) [58].

**Table 3:** Food consumption according to NOVA classification among celiac adults and children (mean of portions/day ± SD).

	Adults			*p* Value		Children and Adolescents			*p* Value		*p* Value (Adults vs. Children)		
	Vt0	Vt3	Vt12	Vt0 vs. Vt3	Vt0 vs. Vt12	Vt0	Vt3	Vt12	Vt0 vs. Vt3	Vt0 vs. Vt12	Vt0	Vt3	Vt12
**n**	27	13	4			31	20	16					
**G1**	6.3 ± 2.4	7.2 ± 2.7	8.2 ± 1.8	NS	0.066	5.6 ± 1.9	6.8 ± 2.0	7.1 ± 2.3	NS	0.078	<0.05	<0.05	NS
**G2**	2.8 ± 1.0	3.2 ± 1.2	3.8 ± 1.3	NS	NS	3.4 ± 1.3	4.4 ± 1.6	3.5 ± 1.0	NS	NS	NS	0.05	NS
**G3**	1.4 ± 1.1	1.5 ± 0.9	1.8 ± 0.6	NS	NS	1.1 ± 0.8	1.1 ± 0.8	1.1 ± 0.9	NS	NS	<0.05	NS	NS
**G4**	5.0 ± 2.2	4.3 ± 1.8	3.7 ± 1.1	<0.05	0.068	6.1 ± 2.1	5.9 ± 2.3	5.9 ± 1.8	NS	<0.05	NS	0.068	<0.05

Abbreviations: G1: Group 1 (unprocessed or minimally processed foods); G2: Group 2 (processed culinary ingredients); G3: Group 3 (processed foods); G4: Group 4 (ultra-processed products). *p* < 0.05: Statistically significant; NS = Not Significant.

**Table 4:** GFP energy contribution to total energy intake among celiac adults and children (mean ± SD).

	Adults			*p* Value		Children and Adolescents					*p* Value (Adults vs. Children)		
	Vt0	Vt3	Vt12	Vt0 vs. Vt3	Vt0 vs. Vt12	Vt0	Vt3	Vt12	Vt0 vs. Vt3	Vt0 vs. Vt12	Vt0	Vt3	Vt12
**n**	27	13	4			25	21	14					
**GFP E%**	23.7 ± 10.0	18.9 ± 6.1	21.4 ± 11.4	<0.05	0.08	17.4 ± 12.6	18.8 ± 14.7	21.4 ± 10.7	NS	NS	<0.05	NS	NS

Abbreviations: GFP: Gluten-free product. *p* < 0.05: Statistically significant; NS = Not Significant.

**Table 5:** FODMAP consumption (g/day) of celiac adults and children (mean ± SD).

	Adults			*p* Value		Children			*p* Value		Adults vs. Children		
	Vt0	Vt3	Vt12	Vt0 vs. Vt3	Vt0 vs. Vt12	Vt0	Vt3	Vt12	Vt0 vs. Vt3	Vt0 vs. Vt12	Vt0	Vt3	Vt12
n	25	12	4			30	21	11					
Fructose	11.3 ±5.5	16.4 ± 12.3	14.7 ± 4.9	0.18	NS	10.2 ± 5.2	11.8 ± 6.3	13.2 ± 6.4	NS	0.06	NS	NS	NS
Glucose	9.5 ± 4.7	14.2 ± 9.5	12.1 ± 4.8	<0.05	NS	8.6 ± 4.2	10.6 ± 5.3	9.9 ± 4.8	NS	NS	NS	NS	NS
Fructans	0.8 ± 1.1	0.8 ± 1.0	13.0 ± 1.0	NS	NS	0.5 ± 0.6	0.9 ± 1.1	0.3 ± 0.5	0.08	NS	NS	NS	NS
Lactose	10.4 ± 7.5	12.0 ± 8.9	13.0 ± 9.5	NS	NS	18.8 ± 10.8	13.8 ± 6.8	14.8 ± 8.6	NS	NS	<0.05	NS	NS
Inulin	0.1 ± 0.2	0.4 ± 0.6	0.3 ± 0.5	0.1	NS	0.4 ± 0.3	0.2 ± 0.2	0.3 ± 0.4	0.055	NS	<0.01	NS	NS
Manitol	0.1 ± 0.2	0.0 ± 0.0	0.1 ± 0.3	NS	NS	0.02 ± 0.1	0.01 ± 0.1	0.04 ± 0.2	NS	NS	NS	NS	NS
Raffinose	NC	NC	NC	-	-	NC	NC	NC	-	-	-	-	-
Stachyose	0.02 ± 0.1	0.0 ± 0.0	0.0 ± 0.0	NS	NS	0.0 ± 0.0	0.0 ± 0.0	0.0 ± 0.0	NS	NS	NS	NS	NS
Sorbitol	0.4 ± 0.6	1.3 ± 1.9	1.4 ± 2.1	<0.01	NS	1.0 ± 1.2	0.7 ± 0.7	0.8 ± 1.1	NS	NS	<0.05	NS	NS
TOTAL FODMAP	32.8 ± 12.1	45.1 ± 26.8	42.3 ± 18.5	<0.05	NS	39.4 ± 15.2	38.2 ± 11.4	41.8 ± 13.5	NS	NS	0.08	NS	NS

Abbreviations: NC, No Comsumption. *p* < 0.05: Statistically significant; NS = Not Significant.

## Supplementary Materials

The following are available online at https://www.mdpi.com/article/10.3390/s22030732/s1, Supplementary Table S1. Anthropometric and biochemical data of celiac adults, Supplementary Table S2. Percentage of consumption of each ultra-proccesed food-group (G4) among celiac adults and children.
