# Supplementary material for: Application of a Platform for Gluten-Free Diet Evaluation and Dietary Advice: From Theory to Practice
Source: Sensors (Basel). 2022 Jan 19;22(3):732. doi: 10.3390/s22030732 (PMC8838721; doi:10.3390/s22030732)
Supplement: Supplementary file 1 [file sensors-22-00732-s001.zip › sensors-1510898-supplementary.pdf]

Supplementary Table S1. Anthropometric and biochemical data of celiac adults.

		Adults					Children and adolescents						Adults vs children		
		**vt0**	**vt3**	**vt12**	***p* value**			**vt0**	**vt3**	**vt12**	***p* value**		***p* value**		
**N**		27	13	3	vt0 *vs* vt3	vt0 *vs* vt12		22	10	4	vt0 *vs* vt3	vt0 *vs* vt12	**Vt0**	**Vt3**	**Vt12**
Anthropometric measurements	*Reference values**						*Reference values**								
**Weight (kg)**		62.4±12.1	64.8±16.7	57.2±12.5	NS	NS		29.7±12.1	30.5±12.1	32.0±12.0	0.002	<0.001	NS	<0.001	<0.05
**Height (m)**		1.7±0.1	1.7±0.1	1.7±0.1	NS	NS		1.28±0.2	1.30±0.2	1.33±0.2	<0.001	<0.001	<0.001	<0.001	<0.001
**BMI (kg/m^2)**<br>**% low**<br>**% normal**<br>**% overweight**<br>**% obese**	 <18.5<br>18.5-24.9<br>25.0-29.9<br>>30.0	22.7±3.7<br>8<br>74<br>11<br>7	22.7±3.5<br>0<br>77<br>23<br>0	21.0±2.4<br>0<br>100<br>0<br>0	NS	NS	 <P3<br>P3-P85<br>P85-P95<br>>P95	16.5±1.8<br>7.4<br>85.2<br>3.7<br>3.7	16.5±1.3<br>13.6<br>77.3<br>4.5<br>4.5	16.7±1.9<br>13.3<br>80<br>0<br>6.7	NS	NS	<0.001	<0.001	<0.05
**WHR**<br>**very low risk**<br>**low risk**<br>**high risk**	 <0.95<br>0.96-0.99<br>>1.00	0.8±0.1<br>69<br>16<br>15	0.8±0.1<br>42<br>50<br>8	0.8±0.1<br>75<br>25<br>0	NS	NS	 <P5<br>P5-P95<br>>P95	0.7±0.1<br>77<br>19<br>4	0.7±0.1<br>76<br>19<br>5	0.7±0.1<br>74<br>13<br>13	NS	NS	<0.001	<0.001	<0.001
**Fat mass (kg)**<br>**% low**<br>**% normal**<br>**% high**	 <8.9/12.9% of BW<br>40% of BW<br>>40.1% of BW	27.5±8.6<br>19<br>48<br>33	26.6±5.3<br>8<br>59<br>33	27.2±3.6<br>0<br>100<br>0	NS	NS	 <P5<br>P5-P95<br>>P95	17.1±6.2<br>20<br>60<br>20	18.0±7.0<br>20<br>60<br>20	16.3±3.4<br>20<br>66.7<br>13.3	NS	NS	<0.001	<0.001	<0.01
**Muscle mass (kg)**<br>**% low**<br>**% normal**<br>**% high**	 <39.9% of BW<br>40% of BW<br>>40.1% of BW	24.8±5.6<br>15<br>78<br>7	25.9±7.9<br>0<br>92<br>8	22.3±3.4<br>0<br>100<br>0	NS	NS	 <41.9% of BW<br>42-47% of BW<br>>47.1% of BW	12.1±4.8<br>38.5<br>61.5<br>0	12.4±4.9<br>23.8<br>76.2<br>0	.2±4.8<br>33.3<br>66.7<br>0	NS	NS	<0.001	<0.001	<0.05
**Protein content (kg)**<br>**% low**<br>**% normal**<br>**% high**	 Personalized calculation of InBody 120	8.8±1.8<br>0<br>100<br>0	9.3±2.6<br>0<br>100<br>0	6.8±2.7<br>0<br>100<br>0	NS	NS	 Personalized calculation of InBody 120	4.8±1.7<br>24<br>76<br>0	6.8±8.5<br>10<br>90<br>0	5.2±1.6<br>33<br>67<br>0	NS	NS	<0.001	NS	NS
**Mineral content (kg)**		3.2±0.6	3.3±0.9	2.9±0.4	NS	NS		1.7±0.6	1.7±0.3	1.9±0.6	NS	NS	<0.001	<0.001	<0.05

**% low**<br>**% normal**<br>**% high**	Personalized calculation of InBody 120	0<br>100<br>0	0<br>100<br>0	0<br>100<br>0			Personalized calculation of InBody 120	8<br>92<br>0	5<br>95<br>0	0<br>100<br>0				
**Water content (L)**		33.0±6.7	34.4±9.6	30.1±4.1	NS	NS		17.9±6.2	25.5±31.7	19.5±6.0	NS	NS	<0.001	<0.001	<0.001
**% low**<br>**% normal**<br>**% high**	Personalized calculation of InBody 120	0<br>100<br>0	0<br>100<br>0	0<br>100<br>0			Personalized calculation of InBody 120	23<br>77<br>0	10<br>90<br>0	27<br>73<br>0				
**Basal Metabolic Rate (kcal)**		1339.7±194.6	1386.8±270.5	1260.0±145.1	NS	NS		901.2±184.2	932.5±196.3	944.1±178.4	0.058	0.001	<0.001	<0.001	<0.05
**Energy expenditure (kcal)**		2182.9±335.1	2226.2±405.9	2025.0±168.4	NS	NS		1677.7±346.6	1740.9±754.3	1862.6±297.6	0.093	0.001	<0.001	<0.05	NS
Biochemical data	**Reference values***						**Reference values***							
**Glucose (mg/dl)**	76-110	89.8±10.5	83.1±7.1	87.0±2.0	NS	NS	76-110	80.3±7.1	86.1±6.4	81.3±9.6	0.027	0.043	<0.05	NS	NS
**Total cholesterol (mg/dl)**	<200	173.9±35.2	168.7±19.2	154.0	NS	NS	<200	155.6±21.7	155.8±22.8	160.7±46.4	NS	NS	NS	NS	NS
**HDL (mg/dl)**	>40	49.6±9.9	59.4±16.0	DNC	NS	NS	>40	50.0±10.3	57.7±18.5	63.2±17.5	NS	NS	NS	NS	NS
**LDL (mg/dl)**	<130	95±2.8	95±2.8	95	NS	NS	<130	98.1±22.0	101.0±20.4	85.3±37.9	NS	NS	NS	NS	NS
**TG (mg/dl)**	<150	136.6±248.8	70.0±29.9	57.0	NS	NS	<150	68.1±31.8	52.8±19.2	56.0±23.5	NS	NS	NS	NS	NS
**Ferritin (ng/ml**	20-200	30.6±30.1	18.8±14.3	15.0	NS	NS	20-200	28.7± 14.6	36.1±22.4	29.1±12.5	NS	NS	NS	NS	NS
**Transferrin (mg/dl)**	200-374	273.7±21.0	310.5±159	239.0	NS	NS	200-374	249.5± 110.9	273.4±73.5	302.3±68.7	NS	NS	NS	NS	NS

Abbreviations: Vt0= visit at time 0, at diagnosis; vt3= visit after 3 months on a gluten-free diet; vt12= visit after 12 months on a gluten-free diet; DNC= Data Not Collected; NS: Not significant; BMI: Body Mass Index; WHR: Waist to Hip Ratio; HDL-c: High Density Lipoprotein –cholesterol; LDL-c: Low Density Lipoprotein-cholesterol; TG: Triglycerides.

*Reference values for adults: For adults: BMI, WHR and Fat Mass for aduts: World Health Organization established limits[41]. For children and adolescents: BMI: Sobradillo *et al*., 2004 [33] established limits; WHR and Fat Mass: Moreno *et al.* 1998 [36]and Moreno *et al.* 1999 [37] established limits. For all participants: Muscle Mass: Heymsfield et al. 1990 [38]and Ito et al. 2001 [39] established limits; Protein, mineral and water content: limits established by InBody 120 for each participant; Biochemical data: Basque Health System established values.

Supplementary Table S2. Percentage of consumption of each ultra-proccesed food-group (G4) among celiac adults and children.

	**Adults**			**Children and adolescents**		
	**Vt0**	**Vt3**	**Vt12**	**Vt0**	**Vt3**	**Vt12**
**n**	27	13	4	31	20	18
**GFP**	51.1	48.5	57.7	54.4	52.0	51.8
**Ultraprocessed dairy products**	5.9	10.7	7.7	10.9	10.5	13.4
**Snacks**	7.6	6.1	3	5.5	6.2	4.2
**Candy and chocolates**	8.6	12.6	18.7	12.6	14.1	8.4
**Ultraprocessed fish**	0.4	0.9	1.4	0.6	0.7	0
**Sausages**	7.6	4.5	4.7	11.5	7.6	14
**Fruit derivatives**	5.9	4	0	1.9	2.3	0.3
**Sauces**	4.1	5	1.4	1.5	2.3	4.9
**Soft drinks**	3.3	3.6	1.4	0.2	0.7	2.7
**Alcohol**	6.1	3.1	3	0	0	0

Abbreviations: GFP: Gluten-Free Products.
